# *Ixora coccinea* Enhances Cutaneous Wound Healing by Upregulating the Expression of Collagen and Basic Fibroblast Growth Factor

**DOI:** 10.1155/2014/751824

**Published:** 2014-01-29

**Authors:** Aadesh Upadhyay, Pronobesh Chattopadhyay, Danswrang Goyary, Papiya Mitra Mazumder, Vijay Veer

**Affiliations:** ^1^Division of Pharmaceutical Technology, Defence Research Laboratory, DRDO, Tezpur, Assam 784001, India; ^2^Department of Pharmaceutical Sciences, Birla Institute of Technology, Mesra, Ranchi, Jharkhand 835215, India

## Abstract

*Background*. *Ixora coccinea* L. (Rubiaceae) has been documented for traditional use in hypertension, menstrual irregularities, sprain, chronic ulcer, and skin diseases. In the present study, *I. coccinea* was subjected to *in vitro* and *in vivo* wound healing investigation. *Methods*. Petroleum ether, chloroform, methanol, and water sequential *I. coccinea* leaves extracts were evaluated for *in vitro* antioxidant, antimicrobial, and fibroblast proliferation activities. The promising *I. coccinea* methanol extract (IxME) was screened for *in vivo* wound healing activity in Wistar rat using circular excision model. Wound contraction measurement, hydroxyproline quantification, and western blot for collagen type III (COL3A1), basic fibroblast growth factor (bFGF), and Smad-2, -3, -4, and -7 was performed with 7-day postoperative wound granulation tissue. Gentamicin sulfate (0.01% w/w) hydrogel was used as reference standard. *Results*. IxME showed the potent antimicrobial, antioxidant activities, with significant fibroblast proliferation inducing activity, as compared to all other extracts. *In vivo* study confirmed the wound healing accelerating potential of IxME, as evidenced by faster wound contraction, higher hydroxyproline content, and improved histopathology of granulation tissue. Western blot analysis revealed that the topical application of *I. coccinea* methanol extract stimulates the fibroblast growth factor and Smad mediated collagen production in wound tissue.

## 1. Background

The World Health Organization estimated that 80% of the world's population still relies on plant-based medicines for their primary health care, and skin related disorders specially wounds, which is the most common reason for medical visits in the developing countries. Wounds and wound management therapy have a long history and in the different Ayurvedic classics of India like Charaka Samhita, Sushruta Samhita, and Ayurveda Siksha approximately 70% of the wound healing medicines are of plant origin. In the last few decades, traditional wound healing plants have received enough attention for scientific investigations [1–3], where pathophysiological process of wound healing and various related activities such as fibroblast growth stimulation and antioxidant and antimicrobial activities has been extensively studied and correlated to the rationale of the traditional plant medicines [1, 4, 5]. In thrust of finding for an effective wound healing herb,   *Ixora coccinea* L. (Rubiaceae) was selected for the *in vitro* and *in vivo* wound healing investigations. *I. coccinea* is a small-medium evergreen shrub, widely cultivated ornamental plant throughout South Asian regions, and, used in the treatment of various ailments like infection, hypertension, menstrual irregularities, sprain, chronic ulcer, and skin diseases including cutaneous wounds [6–8]. The plants have been reported for cytotoxic, antitumor, antimicrobial, and anti-inflammatory activity [7, 9–13]. The earlier reports of *I. coccinea* indicated the preliminary wound healing and antimicrobial properties of flower and root extracts [6, 8], but the effect of *I. coccinea* leaf was not properly investigated on various aspects of wound healing. Therefore, the present study was aimed to investigate the effect of *I. coccinea* leaves extract on fibroblast proliferation and related growth factors involved in collagen production pathway in wound granulation tissue.

## 2. Materials and Methods

### 2.1. Cell Line, Bacterial Culture, and Chemicals

Human dermal fibroblast (HDF10605) cell line was procured from Sigma Aldrich. The bacterial strains *Bacillus subtilis* (MTCC 441), *Staphylococcus aureus* (MTCC 3160), *Streptococcus mutans* (MTCC 890), *Escherichia coli* (MTCC 443), *Klebsiella pneumoniae* (MTCC 109), and *Pseudomonas aeruginosa* (MTCC 741) were obtained from Institute of Microbial Technology (IMTECH), Chandigarh. Mueller-Hinton broth, butylated hydroxytoluene (BHT), gallic acid, 2,2-diphenyl-1-picrylhydrazyl scavenging (DPPH), and nitroblue tetrazolium (NBT) were purchased from Sigma. The Folin-Ciocalteu reagent, phenazine methosulfate (PMS), *β*-nicotinamide adenine dinucleotide (NADH), potassium ferrocyanide, trichloroacetic acid (TCA), ferric chloride, hydrogen peroxide (H_2_O_2_), phenazine methosulfate fluoride (PMSF), dimethyl sulphoxide (DMSO), and 3-(4,5-dimethylthiazol-2-yl)-2,5-diphenyltetrazolium bromide salt (MTT) purchased were from Himedia (Mumbai, India). All primary and secondary antibodies and BCIP (5-bromo-4-chloro-3′-indolyphosphate p-toluidine salt)-NBT reagent were purchased from Santa Cruz Biotech, Inc. (Texas, USA). The solvents and chemicals which are not mentioned in the text were of analytical grade.

### 2.2. Preparation of Extracts

*I. coccinea* leaves were collected during September-October (2011) from campus garden of Defence Research Laboratory, Tezpur, Assam, (India), and authenticated at Botanical Survey of India, Shillong (India), and the specimen sample deposited (Acc. no. 081168). About 100 g of shade dried leaves powder was successively extracted with petroleum ether, chloroform, methanol, and water at 1500 lb at room temperature in Accelerated Solvent Extractor (ASE 1.5, Dionex, USA). The extraction was considered complete when the initial color of the percolate gradually changed to colorless. Each solvent extract was concentrated in rotary evaporator (Rotavac, Heidolph2, Germany) under reduced pressure and the water extract was freeze-dried. Preliminary phytochemical screening was performed as described earlier [14].

### 2.3. Antimicrobial and Antioxidant Wound Healing Relevant Assays

Agar broth dilution technique was used in the determination of minimum inhibitory concentration (MIC) in antimicrobial screening according to Hayouni et al. [15].

Antioxidant evaluations including DPPH free radical and superoxide anion radical scavenging activity (SRSA), ferric ion reducing antioxidant power (FRAP), and total phenolic content were performed as described earlier [16–19].

### 2.4. Fibroblast Proliferation Assay

Human dermal fibroblast cells were cultured in DMEM containing 10% Fetal Bovine Serum (FBS) and antibiotics (100 U/mL penicillin and 100 U/mL streptomycin) in a humidified CO_2_ incubator with 5% CO_2_ at 37°C. Media were replaced every alternate day. Cells were harvested on confluency, using 0.05% Trypsin-EDTA and subcultured in fresh media for producing single cell suspension.

The fibroblast proliferation assay was performed as described by Adetutu et al. [4]. The cells were counted using a Scepter 2.0 Automated Cell counter (Millipore, USA) and seeded at a density of 1 × 10^3^ cells per well in 96-well plate excluding the first row and maintained at 37°C in humidified 5% CO_2_ atmosphere with 10% FBS. The medium was replaced after 24 h incubation with DMEM containing 0.5% FBS and extract samples. All samples were dissolved in DMSO. The final concentrations of extracts ranged from 1.56 to 100 *μ*g/mL and for DMSO 0.4% v/v. DMEM/0.5% FBS (with DMSO) and DMEM/10% FBS serve as normal control and growth stimulation controls. After 48 h of incubation cell viability was assayed by MTT assay method. The MTT solution (5 mg/mL) was added to each well before 4 h of culture termination. DMSO was added to each well and optical density was measured at 570 nm, using a microplate reader (SpectraMax Plus 384, Molecular Devices, USA). Each sample was assayed in triplicate and three independent tests were performed.

### 2.5. Hydrogen Peroxide Induced Oxidative Stress

Hydrogen peroxide (1.0 × 10^−4^ M) was used to induce oxidative stress as described by Annan and Houghton [5]. Dermal fibroblast cells were seeded in 96-well plates (5 × 10^3^ cells/well) containing DMEM/10% FBS, incubated at 37°C in humidified 5% CO_2_ atmosphere. After 24 h the growth medium was replaced with fresh DMEM containing different concentrations of extracts (1.56–100 *μ*g/mL) and simultaneously exposed to 1.0 × 10^−4^ M hydrogen peroxide and incubated for 3 h at 37°C. Catalase (250 U/mL) was used as positive control. After the incubation, cell viability was assessed by MTT assay method. Each sample was assayed in triplicate and three independent tests were performed.

### 2.6. Wound Healing Activity

#### 2.6.1. Animals

Healthy adult Swiss albino male mice (20–25 g) and male Wistar rats (250–300 g) housed in Defence Research Laboratory (DRL), Tezpur, Assam (India), were acclimatized for 3 days. They were given free access to food and water *ad libitum*. The experiments were performed according to the Institutional Animal Ethical Committee guidelines (IAEC/DRL/05/July/2011) of DRL, Tezpur.

#### 2.6.2. Acute Skin Irritation and Toxicity Study

The acute skin irritation and toxicity study was performed for 1% and 2.5% (w/w) IxME hydrogel according to the OECD guidelines 402 (OECD guidelines, 1987) [20]. Hydrogel was applied on the shaved back of the mice and monitored for 14 days for abnormal skin response including irritation, redness itching, inflammation, and other related symptoms.

#### 2.6.3. Animal Grouping and Excision Wound Creation

All Wistar rats were anesthetized with sodium phenobarbitone (40 mg/kg) intraperitoneally (i.p.). Circular 20 mm diameter wounds were caused on dorsal skin of each animal up to the depth of loose subcutaneous tissue using surgical scissor and forceps. Animals were randomly divided into four groups: nontreated (group I), vehicle control (Carbopol 934 containing 5% propylene glycol, group II), *I. coccinea* methanol extract (IxME, 2.5% w/w, group III), and gentamicin sulfate (0.01% w/w, group IV). Each group contains 20 animals and hydrogel formulations were applied topically once daily until complete epithelialization. On the 7th postoperative day, one-third of animals were euthanized and wound granulation tissues (excluding any underlying muscle and extraneous tissue) were harvested. A portion of harvested tissue was immediately stored in liquid nitrogen for further analysis and another portion was fixed in 4% formaldehyde for histopathological evaluations. Half of the remaining animals were euthanized on day 15 after injury; the entire granulation tissue was used for histopathological assessment and remaining animals were observed until complete epithelialization [21].

#### 2.6.4. Wound Contraction Rate and Hydroxyproline Content Estimation

The progressive changes of wounded area were photographed (Nikon Coolpix-S3000 camera) and evaluated by using special size analysis software—ImageJ (National Institutes of Health, Maryland, USA)—as described earlier [21].

Hydroxyproline content was analyzed on day 7 after injury granulation tissue as described by Upadhyay et al. [21]. Tissue hydrolysate samples were mixed with 1 mL of 10 mM CuSO_4_ followed by the addition of 1 mL of 2.5 N NaOH and then 1 mL of 6% H_2_O_2_. The solution was mixed and incubated at 80°C for 5 min with frequent vigorous shaking. Upon cooling, 4 mL of 3 N H_2_SO_4_ was added with agitation. Finally, 2 mL of 5% *p*-dimethyl amino benzaldehyde was added and incubated at 70°C for 15 min. Absorbance was measured at 500 nm using a UV-VIS spectrophotometer (CE7200, CECIL, USA).

#### 2.6.5. Histopathological Evaluation

Granulation tissues were sectioned (6 *μ*m thick) and stained with hematoxylin-eosin (HE) and Masson's trichrome (MT) stains. Tissues sections were examined for epithelialization, inflammatory cell infiltration, fibroblast proliferation, neovascularization, and collagen deposition.

#### 2.6.6. Western Blot Analysis

Western blot analysis was performed as described by Upadhyay et al. [21]. Protein concentration was estimated in tissue homogenate using Bradford reagent (Sigma, Germany). Primary antibody COL3A1-mouse monoclonal IgG_1_ (sc-271249), bFGF-mouse monoclonal IgG_2a_ (sc-74413), Smad 2-goat polyclonal IgG (sc-6200), Smad 4-rabbit polyclonal IgG (sc-7154), Smad 7-mouse monoclonal IgG_1_ (sc-365846), *β*-Actin-mouse monoclonal IgG_1_ (sc-47778), and respective secondary antibody goat anti-mouse IgG-AP (sc-2008), rabbit anti-goat IgG-HRP (sc-2768), and goat anti-rabbit IgG-AP (sc-2007) were purchased from Santa Cruz Biotech. (USA). Smad 3-rabbit polyclonal IgG (Cat-10832) was purchased from Cayman Chemicals (USA). Equal amount of protein was electrophoresed on 12% SDS-PAGE with 4% stacking gel (Mini Trans-Blot, BioRad Laboratories Inc., USA) at 80 V for 45 min. Proteins were transblotted onto the PVDF membrane (Millipore Corp., USA), and processed with COL3A1, bFGF, Smad-2, -3, -4, -7 and *β*-Actin primary antibodies (1 : 1000) and corresponding secondary antibodies (1 : 2000). The desired proteins were detected by BCIP-NBT solution and western Max-HRP-Chromogenic detection kit (Amresco, USA). *β*-actin was estimated as internal control.

### 2.7. Statistical Analysis

The results are expressed as means ± standard deviation (S.D.). Data were statistically analyzed using Dunnett test. A *P* value <0.05 was considered statistically significant as compared to nontreated and vehicle treated group.

## 3. Results

### 3.1. Extract and Phytochemicals

The yield of the* I. coccinea* petroleum ether (IxPE), chloroform (IxCE), methanol (IxME), and water (IxWE) was found 3.31, 2.22, 16.33 and 10.92% (w/w), respectively. The preliminary phytochemical screening of *I. coccinea* extracts showed the presence of alkaloid, flavanoids, terpenes, phenolic, carbohydrate, and saponins in different extracts.

### 3.2. Antimicrobial and Antioxidant Properties

In the antimicrobial assay, methanol extract was found very active (MIC 0.125–2 mg/mL) as compared to other solvent extracts, where the gram positive *B. subtilis* and gram negative *K. pneumoniae* were found the most sensitive (MIC 0.125 and 0.25 mg/mL, resp.). For chloroform extract MIC values were ranging between 1 and 2 mg/mL. Petroleum ether and water extracts showed poor antimicrobial activity against selected pathogens (Table 1).

**Figure 1:**
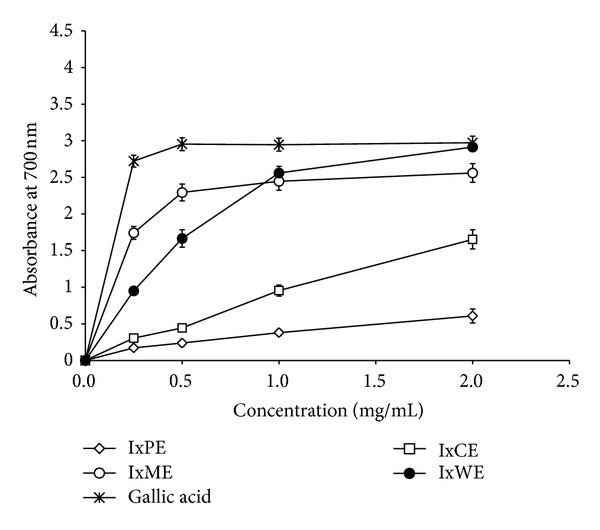
Ferric ion reducing antioxidant power (FRAP) of different *I. coccinea* extracts.

On the other hand, in DPPH, superoxide radical scavenging activity (SRSA), IxPE, and IxCE were found inactive and showing 0.5 and 6.83 gallic acid equivalents, respectively, in FCR assay (Table 2). IxME and IxWE were potent antioxidant extracts, in which IxME showed higher DPPH scavenging property in comparison to IxWE with IC_50_ (*μ*g/mL) values 9.63 and 26.01, respectively. On the other hand, IxWE (IC_50_ = 655.06 *μ*g/mL) possess the higher superoxide radical scavenging strength than IxME (IC_50_ = 838.03 *μ*g/mL). FRAP and FCR assay data further revealed the antioxidant potency of the IxME and IxWE (Figure 1 and Table 2).

### 3.3. Fibroblast Proliferation and Viability

**Figure 2:**
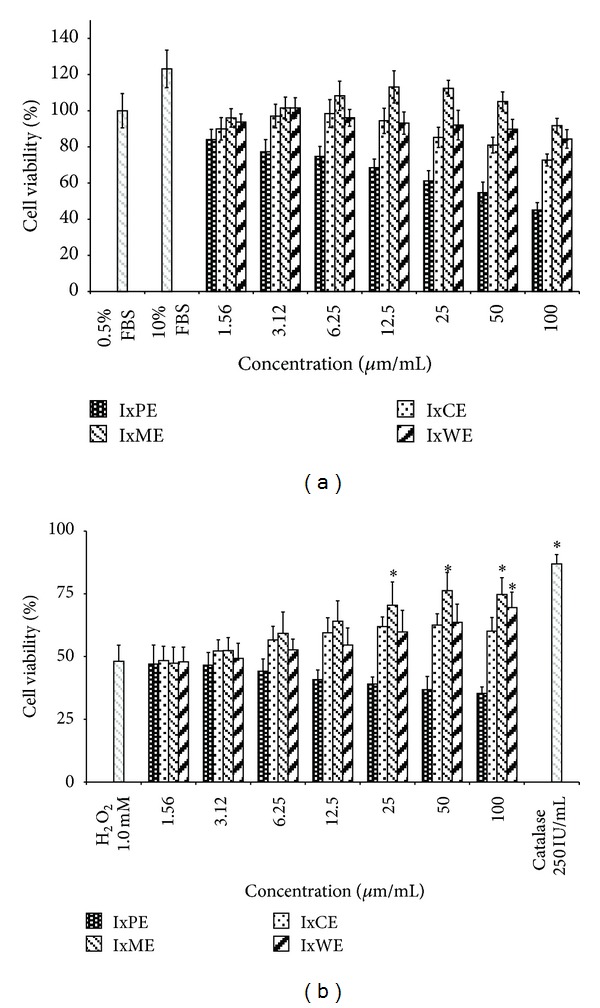
(a) Effect of *I. coccinea* extracts on human dermal fibroblast proliferation. (b) Protection of human dermal fibroblast cells against H_2_O_2_-induced damage with simultaneous application of different *I. coccinea* extracts. FBS: Fetal Bovine Serum; Cat-Catalase. Values are expressed as mean ± SD. Asterisk (∗) indicates significantly different (*P* < 0.05) as compared to H_2_O_2_ treated negative treated groups.

Among all the extracts, IxPE decreased the cell viability from 84.02 to 44.99% in a concentration dependant manner (1.56–100 *μ*g/mL) (Figure 2(a)). The IxCE, IxME, and IxWE extracts showed the biphasic proliferation response, where the cell viability increased at low concentrations and decreased at higher concentrations. The IxCE exposure for 48 h increased the cell viability from 89.95 to 98.55% (1.56 to 6.25 *μ*g/mL), however decreased at higher concentrations (Figure 2(a)). The IxME showed the significant fibroblast proliferation activity, which was almost close to the level of positive control (113.16% at 12.5 *μ*g/mL). On the other hand, IxWE decreased the cell viability from 101.52 to 84.33% (3.12 to 100 *μ*g/mL).

The exposure of H_2_O_2_ (1.0 × 10^−4^ M) for 3 h decreased the cell viability (48.12%). IxPE synergies the effect of H_2_O_2_ and decreased the cell viability from 47.03 to 35.32%, with increasing concentration (Figure 2(b)). Potent antioxidant IxME and IxWE showed dose dependent protection (47.33–74.71% and 47.85–69.47%, resp.). However, IxCE showed 48.36–60.09% cell viability at 1.56–100 *μ*g/mL.

### 3.4. Wound Healing

#### 3.4.1. Acute Skin Irritation and Toxicity

In skin irritation and toxicity assay, any sign of inflammation and irritation were not observed in both 1 and 2.5% (w/w) IxME hydrogel. Therefore, high concentration 2.5% (w/w) IxME hydrogel was selected for *in vivo* wound healing study.

#### 3.4.2. Wound Contraction

**Figure 3:**
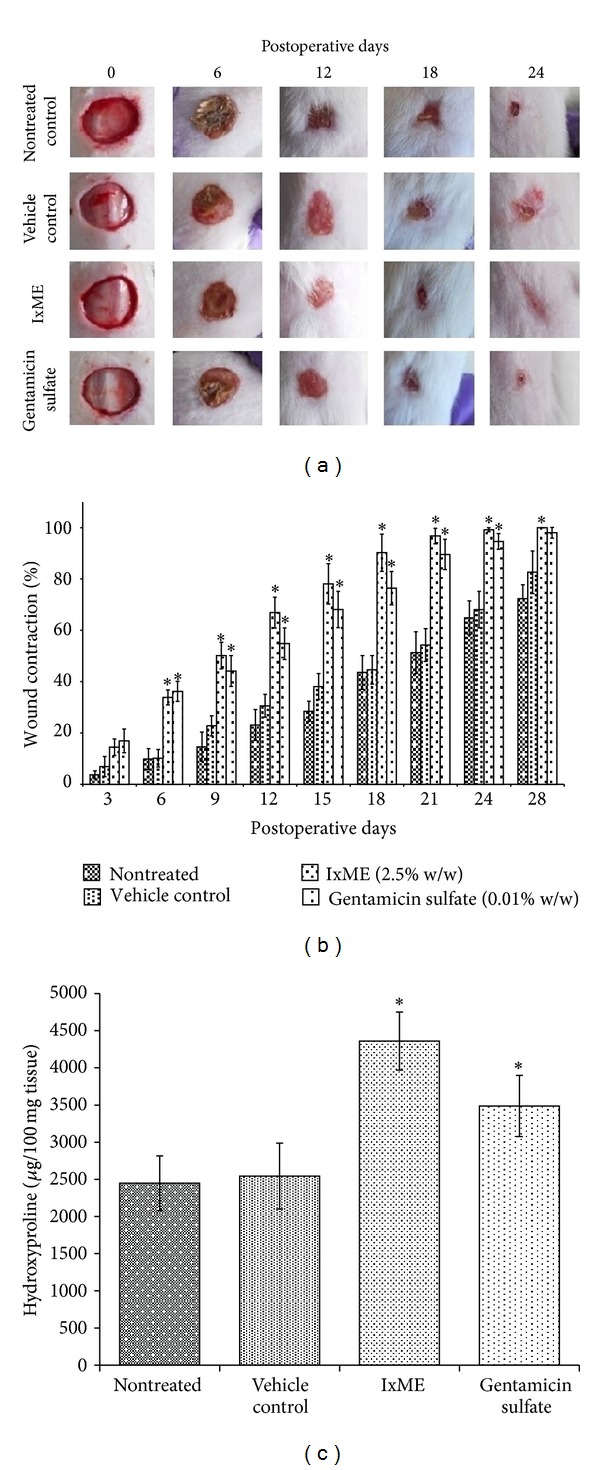
Effect of *I. coccinea* methanol extract (IxME) on wound healing. (a) Pictorial representation of wound closure in wistar rat. (b) Wound contraction rate. (c) Hydroxyproline content. Values are expressed as mean ± SD. Asterisk (∗) indicates significantly different (*P* < 0.05) as compared to the nontreated and vehicle treated groups.

The whole wound area was reduced in parallel to postwound days (Figure 3(a)). Nontreated and vehicle treated animal groups showed 51.3% and 54.25% wound contraction on 21st postoperative days, respectively. On the other hand, IxME treated animal group showed 96.78% and gentamicin sulfate treated group 89.59% wound contraction. The contraction rate was significantly higher in IxME treated group as compared to nontreated and vehicle treated control group.

#### 3.4.3. Hydroxyproline Content

IxME and gentamicin sulfate treated group showed significantly increased level of hydroxyproline as compared to nontreated and vehicle treated groups (Figure 3(b)). Although the hydroxyproline content of IxME treatment group was higher as compared to gentamicin sulfate treated group but the data were statistically not significant.

#### 3.4.4. Histopathological Observations

**Figure 4:**
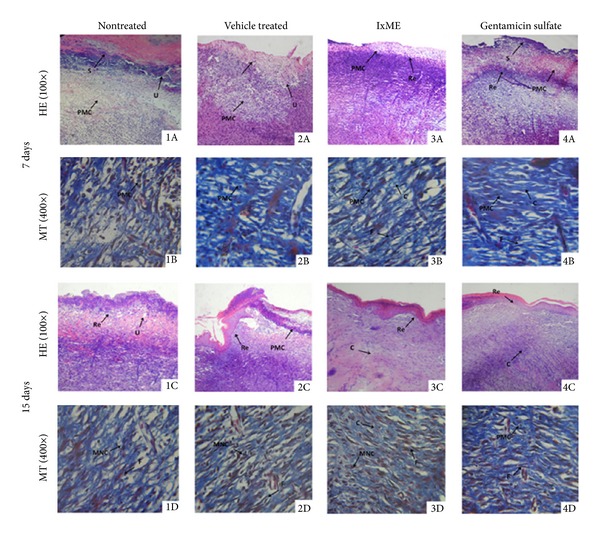
Microscopic view of healing wound granulation tissue and remodeling epidermis/dermis in (1) nontreated, (2) vehicle control, (3) IxME, and (4) gentamicin sulfate treated animal groups. Section shows the hematoxylin and eosin stained epidermis and dermis in (A) and (C) (100x) and Masson's trichrome stained dermis in (B) and (D) (400x) of 7- and 15-day postoperative treated animal groups, respectively. The arrows point to the events of wound healing—S: scab; U: ulcer; Re: reepithelization; F: fibroblast; PMC: polymorphonuclear cells; MNC: mononuclear cells; C: collagen; and NV: neovascularization.

IxME and gentamicin sulfate treated animal groups showed well organized wound healing processes (inflammation, proliferation, and remodeling) in postoperative days. On the other hand, vehicle and nontreated animal groups depict slow rate of epithelialization and dermal layer with lesser collagen bundles (Table 3). Histopathological section of day 7 showed mild to moderate edema and ulcer in nontreated and vehicle treated groups, with abundant polymorphonuclear cell (PMC). The infiltration rate of mononuclear and fibroblast cells were observed low (Figure 4). However, 7-day IxME treated wound tissue showed increased density of mononuclear cells with distinct onset of reepithelialization. Edema and ulcerous area were absent in both IxME and gentamicin sulfate treatments. 15-day IxME treatment significantly accelerated the cutaneous wound healing as depicted by thick well organized re-epithelialized layer, dermis with compact collagen layering, and faster keratinization with intraepithelial cornification, whereas slow reepithelialization with minor ulcer area was noticed in nontreated animal group. Masson's trichrome (400×) staining of 7- and 15-day postoperative wound granulation tissue depicted the clearer picture of wound healing, where IxME treatment showed increased macrophage and fibroblast density with higher collagen deposition. On the other hand, gentamicin treatment although showed complete reepithelialization with irregular packing of collagen fibers and minor to moderate macrophages infiltration (Figure 4). In vehicle and nontreated animal groups, collagen bundles were loosely packed and granulation tissues were moderately cellular with mononuclear and fibroblast cells.

#### 3.4.5. Effect of IxME on Granulation Tissue Protein

**Figure 5:**
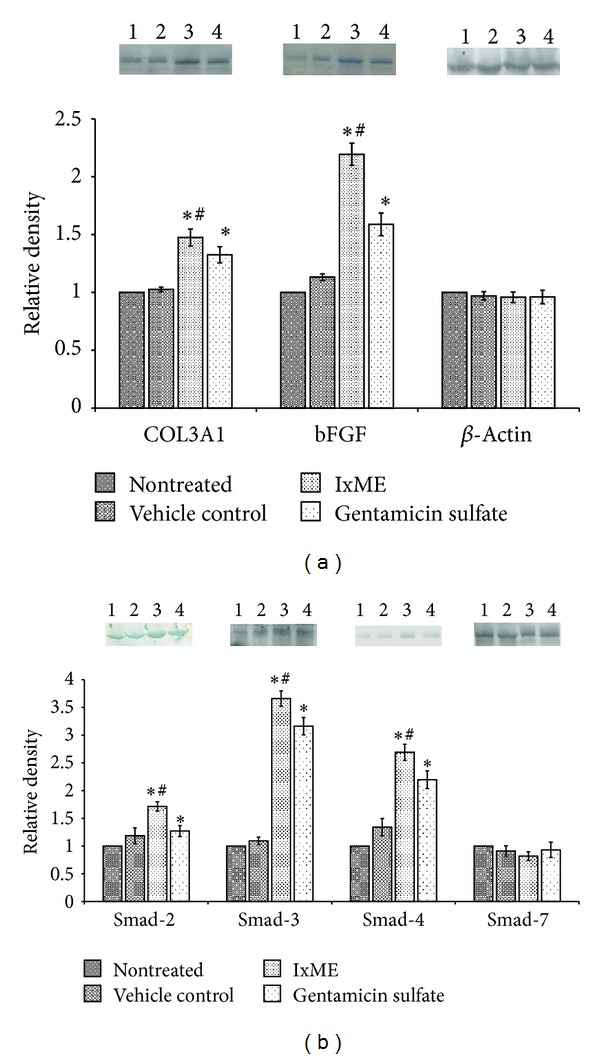
Effect of *I. coccinea* methanol extract (IxME) on COL3A1 and bFGF Smad-2, -3, -4, and -7 protein expressions on day 7 (seven) in wound tissues, detected by western blot. Lane (1) nontreated, (2) vehicle control, (3) IxME, and (4) gentamicin sulfate treated animal group, respectively. Values are expressed as mean ± SD. Asterisk (∗) indicates significantly different (*P* < 0.05) as compared to the nontreated and vehicle treated groups. Hash (#) indicates significantly different (*P* < 0.05) as compared to gentamicin sulfate treated group.

Western blotting showed significantly increased expression of COL3A1 and bFGF protein in IxME and gentamicin sulfate treated wound granulation tissue (Figure 5). The expression levels of signal transducer protein Smad-2, -3, and -4 were also significantly increased, whereas the inhibitory protein Smad-7 was found unaltered. Moreover, the expression levels of the COL3A1, bFGF, and Smad proteins (2, 3, and 4) were significantly higher in IxME treated animals groups in comparison of gentamicin sulphate treated group. The *β*-actin was used as an internal control.

## 4. Discussion

Cutaneous wound healing is a complex cascade of tissue regenerative and restorative events including chemotaxis, cell division, neovascularization, synthesis, and maturation of new extracellular matrix and remodelling of scar. These events can be broadly categorized into inflammation, proliferation, and remodeling phases of wound healing, which are regulated by several mediators including cytokines and various secreted growth factors.

Although, the wound healing cascade takes place by itself and does not require much help, but various risk factors such as infection have serious impact. *B. subtilis*, *S. aureus*, *E. coli*, *K. pneumoniae*, and *P. aeruginosa* are the most wound infecting pathogens, as the open wound provides the favorable conditions for microbial growth [22]. Our study indicated the antimicrobial potency of *I. coccinea* methanol extract (IxME) against most common wound infecting pathogens, for example, *B. subtilis* (MIC = 0.125 mg/mL), *K. pneumoniae* (MIC = 0.25 mg/mL), and *P. aeruginosa* (MIC = 0.50 mg/mL) as compared to other solvent extracts (Table 1), corroborating with the previous reports of antimicrobial potential [8, 11, 13].

Invading microbes prolonged the inflammatory phase of wound healing by producing toxins and wound exudates and subsequently delayed the granulation tissue formation [22]. Activated polymorphonuclear cells including neutrophiles, leukocytes and mononuclear cell (MNC) like lymphocytes, monocytes, and macrophages phagocytize, and kill all the infecting pathogen. Reactive oxygen species (ROS) are the key product for bactericidal activity of these activated cells. ROS like HO^−2^, HO^−^, and O^2−^, although in low concentrations sterile the wound fibrin clot matrix and are the important determinant of wound angiogenesis [23]. Whereas, the profuse ROS not only damage extracellular structure proteins, lipids, and DNA but also stimulate signal transduction pathways to prolong the inflammatory phase of wound healing. Many scientific publications have proven the beneficial effect of different plant-based antioxidants on wound repair process [24, 25] and our findings of free radical scavenging activity (DPPH, SRSA, FRAP, and FCR) in methanol and water *I. coccinea* extracts revealed that this plant may be useful in wound healing (Figure 1 and Table 2).

The proliferation phase of wound healing involves formation of granulation tissue, synthesis and deposition of collagen fibers, and reepithelialization. In the late phase of inflammation, the activated macrophages initiate the proliferation phase, that actively progressed by the infiltrating fibroblasts. Fibroblast cells synthesize the collagen fibers and other cytoskeleton matrix components [26, 27]. *In vitro* fibroblast proliferation assay for *I. coccinea* revealed the toxic effect of IxPE and IxCE as the cell viability decreased (<80% to 0.5% FBS) with increasing concentrations (Figure 2(a)). IxPE decreased cell population could be due to the presence of proliferation inhibitory components in *I. coccinea* petroleum ether extract, which also synergized the H_2_O_2_-induced fibroblast cell death. Whereas, IxCE at 100 *μ*g/mL decreased the cell population below 80% was considered as cytotoxic. In the biphasic proliferation response of IxCE, IxME, and IxWE, the inhibitory effect at higher concentrations might be due to accumulation of growth inhibitory components of respective crude extract (Figure 2(a)). IxME and IxWE showed the concentration dependent protection and effectively antagonized the H_2_O_2_-induced cell death (Figure 2(b)) and this protective effect might be due to antioxidant properties of the extracts (Table 2). The above findings of *in vitro* fibroblast proliferation and protection against H_2_O_2_ coincide with the previous reports of different medicinal plants [1, 3–5]. However, the IxCE did not show any *in vitro* antioxidant activity (DPPH, SRSA, andFRAP); thereby the protective effect against H_2_O_2_ induced oxidative stress could be due to the intracellular antioxidant enzymes (superoxide dismutase, glutathione peroxidase, catalase, etc.) inducing activity.

From the above *in vitro* antimicrobial, antioxidant, and fibroblast cell proliferation studies it was observed that *I. coccinea* methanol extract possesses the highest activity as compared to other extracts. Keeping in view of the above findings, IxME (2.5% w/w) was further evaluated for *in vivo* wound healing activity in Wistar rat in circular excision model.

In wound repair process, the centripetal movements of surrounding epithelial tissues (rallied by the maturing extracellular matrix) close the wound opening [27]. Collagen is a major component of extracellular matrix and wound repair process depends on the regulated production and deposition/maturation of new collagen. Hydroxyproline is a basic constituent of collagen structure and its content is an index of collagen synthesis. The periodic assessment of wound area showed that the topical application of IxME significantly accelerated the wound contraction rate and hydroxyproline content as compared to nontreated and hydrogel vehicle treated groups. The wound contraction rate for IxME was even faster than gentamicin treated group, although the data was not significant (Figure 3). Fibroblasts in granulation tissue regulate the production, deposition, and their subsequent maturation of collagen fibers that impart physical strength to the tissue. Histopathological examination of IxME treated tissue on 7 and 15 postoperative days showed the well organized wound healing with prominent macrophage and fibroblast infiltration. Reepithelialization was also higher with perfuse collagen deposition in mature dermis as compared to nontreated and vehicle control, both (Figure 4).

During proliferation phase, various secreted chemotactic molecules and growth factors such as transforming growth factor-*β* (TGF-*β*), basic fibroblast growth factor (bFGF), and platelet derive growth factor (PDGF) control the motion of macrophage/fibroblasts [27, 28]. bFGF promotes the neovascularization in granulation tissue to increase oxygen supply that facilitates the collagen production and maturation. Released TGF-*β* bounds to the extracellular fibroblast TGF-*β* receptors and initiates the TGF-*β*-Smad mediated collagen production. A complex interplay of Smad family proteins (Smad-2, -3, -4, and -7; TGF-*β* type I receptor kinases substrate) transduced the receptor signals to specific target gene and regulate the synthesis of collagen in granulation tissue [28, 29]. The mechanism of collagen production through Smad mediated signaling pathway in the granulation fibroblast has been revealed earlier [22, 28, 29] and the present findings of western blot analysis showed that IxME hydrogel topical application significantly upregulated the expression of bFGF, COL3A1, and Smad-2 and -4 proteins as compared to gentamicin sulfate and control groups, whereas the expression of Smad-7, the inhibitory protein was unaltered (Figure 5). Histopathological examination and expression of tissue protein (bFGF and COL3A1) supported the *in vitro* findings of fibroblast proliferation potential of IxME.

## 5. Conclusion

The present study revealed that *I. coccinea* methanol extract mediated wound healing activity may be a combine effect of antimicrobial, antioxidant and fibroblast proliferating properties, which is also supported by the *in vitro* and *in vivo* experimental studies.

## Figures and Tables

**Table 1:** Antibacterial activity of different *I. coccinea* leaves extracts expressed as minimal inhibitory concentration (MIC) in mg/mL.

Bacteria	MIC (mg/mL)			
	IxPE	IxCE	IxME	IxWE
*Bacillus subtilis *(MTCC111)	2	>2	0.125	1
*Staphylococcus aureus *(MTCC3160)	>2	>2	1	>2
*Streptococcus mutant* (MTCC890)	>2	1	2	2
*Escherichia coli *(MTCC443)	2	1	2	>2
*Klebsiella pneumoniae *(MTCC109)	>2	>2	0.25	>2
*Pseudomonas aeruginosa* (MTCC741)	2	2	0.5	2

IxPE: *I*.* coccinea *petroleum ether extract; IxCE: *I*.* coccinea *chloroform extract; IxME: *I*.* coccinea *methanol extract; IxWE: *I*.* coccinea *water extract. Chloramphenicol was used as positive control (MICs < 90 *μ*g/mL).

**Table 2:** Scavenging activity of different *I. coccinea* extracts against DPPH and superoxide anion free radicals and their Folin-Ciocalteu reagent (FCR) reducing capacity.

*Ixora coccinea *	Scavenging activity against DPPH radical (IC_50_ ± S.D.)	Scavenging activity against superoxide radical (IC_50_ ± S.D.)	FCR reducing capacity (mg_GAc_/g_extract_)
IxPE	NA	NA	0.5 ± 0.04
IxCE	NA	NA	6.83 ± 0.5
IxME	9.63 ± 1.22	838.03 ± 21.04	27.75 ± 0.55
IxWE	26.01 ± 2.66	655.06 ± 17.83	12.32 ± 0.43
Gallic acid	1.10 ± 0.39	156.86 ± 36.39	—

IC_50_ values are expressed in *μ*g/mL; NA: not active; S.D.: standard deviation; IC_50_: amount of antioxidant necessary to scavenge the initial DPPH/superoxide radical by 50%; mg_GAc_/g_extract_: mg of gallic acid equivalent/gram of extract; GAc: gallic acid; IxPE: *I*.* coccinea *petroleum ether extract; IxCE: *I*.* coccinea *chloroform extract; IxME: *I*.* coccinea *methanol extract; IxWE: *I*.* coccinea *water extract.

**Table 3:** Histopathological evaluation of wound healing process in different treatment groups.

Groups	Wound healing process							
	S	U	Ed	PMC	MNC	FP	RE	CD
Day 7								
Nontreated	++	++	++	+++	+	+	−	−
Vehicle control	++	++	++	+++	+	+	+/−	−/+
IxME (2.5%)	+	−	−	++	++	+++	++	++
Gentamicin sulfate (0.01%)	+	−	−	++	++	++	+	+
Day 15								
Nontreated	++	+	−/+	++	+++	++	+	++
Vehicle control	+	+	−	++	+++	++	++	++
IxME (2.5%)	−	−	−	−	+	+++	+++	+++
Gentamicin sulfate (0.01%)	−	−	−	−/+	+	++	++	++

HE and MT staining were scored as mild (+), moderate (++), and severe (+++) for epidermal and/or dermal re-modeling. S: scab; U: ulcer; Ed: edema; PMC: polymorphonuclear cells; MNC: mononuclear cells; FP: fibroblast proliferation; CD: collagen deposition; RE: reepithelialization; IxME: *I. coccinea* methanol extract.
